# Associations of Sedentary Time and Physical Activity From Childhood With Lipids: A 13-Year Mediation and Temporal Study

**DOI:** 10.1210/clinem/dgad688

**Published:** 2023-12-14

**Authors:** Andrew O Agbaje

**Affiliations:** Institute of Public Health and Clinical Nutrition, School of Medicine, Faculty of Health Sciences, University of Eastern Finland, Kuopio 70211, Finland; Children's Health and Exercise Research Centre, Department of Public Health and Sports Sciences, Faculty of Health and Life Sciences, University of Exeter, St Luke’s Campus, Heavitree Road, Exeter EX1 2LU, UK

**Keywords:** pediatrics, dyslipidemia, causal inference, longitudinal study, movement behavior, body composition, lifestyle modification

## Abstract

**Context:**

Among children, evidence on long-term longitudinal associations of accelerometer-measured sedentary time, light physical activity (LPA), and moderate to vigorous PA (MVPA) with lipid indices are few. The mediating role of body composition and other metabolic indices in these associations remains unclear and whether poor movement behavior precedes altered lipid levels is unknown.

**Objective:**

This study examined the associations of sedentary time, LPA, and MVPA from childhood through young adulthood with increased lipids, the mediating role of body composition, and whether temporal interrelations exist.

**Methods:**

Data from 792 children (58% female; mean [SD] age at baseline, 11.7 [0.2] years), drawn from the Avon Longitudinal Study of Parents and Children (ALSPAC) UK birth cohort, who had at least 2 time-point measures of accelerometer-based sedentary time, LPA, and MVPA during clinic visits at ages 11, 15, and 24 years and complete fasting plasma high-density lipoprotein cholesterol, low-density lipoprotein cholesterol, triglyceride, and total cholesterol measured during follow-up visits at ages 15, 17, and 24 years were analyzed.

**Results:**

Total fat mass partly mediated the inverse associations of LPA with low-density lipoprotein cholesterol by 13%, triglyceride by 28%, and total cholesterol by 6%. Total fat mass mediated the inverse associations of MVPA with low-density lipoprotein cholesterol by 37% and total cholesterol by 48%, attenuating the effect on total cholesterol to nonsignificance (*P* = .077). In the temporal path analyses, higher MVPA at age 15 years was associated with lower low-density lipoprotein cholesterol at 24 years (β = −0.08, SE, 0.01, *P* = .022) but not vice versa.

**Conclusion:**

Sedentary time worsens lipid indices, but increased LPA had a 5- to 8-fold total cholesterol-lowering effect and was more resistant to the attenuating effect of fat mass than MVPA.

Elevated lipid levels and dyslipidemia in childhood have been associated with subclinical atherosclerosis in midadulthood and premature cardiovascular mortality in midlife (1, 2). Emerging longitudinal studies suggest that elevated lipid and dyslipidemia's effect on subclinical atherosclerosis may be evident in young adulthood and that early intervention in late adolescence may reverse atherosclerotic processes (3, 4). A 20-year long-term dietary counseling randomly assigned but unmasked group trial conducted among 1116 infants from 5 months until age 20 years with a 6-year postintervention follow-up reported minimal or no statistically significant difference in total cholesterol, high-density lipoprotein cholesterol, low-density lipoprotein cholesterol, and triglyceride in the intervention and control group (5). Moreover, the observed positive physical activity (PA) effects on lower lipids levels during clinical trials tend to significantly attenuate after the intervention period and the reason remains unclear (6, 7).

The recent World Health Organization PA guideline that recommended decreasing sedentary time and increasing moderate to vigorous PA (MVPA) in children and adolescents for the prevention of cardiometabolic diseases was based largely on cross-sectional reports that accelerometer-based PA was associated with lower triglyceride and higher high-density lipoprotein cholesterol (8, 9). However, long-term longitudinal evidence on the associations of accelerometer-measured sedentary time and MVPA with lipid indices in children are few, and existing ones are of low quality (6-8, 10). This PA guideline did not specifically mention light PA (LPA) for children and adolescents due to scarce evidence (8). Recent longitudinal reports suggest that LPA maybe more effective in lowering inflammation than MVPA (11). Longitudinal evidence on accelerometer-measured LPA in association with lipid indices is limited in the pediatric population (6-8). The longitudinal mediating roles of body composition, insulin resistance, and inflammation in these associations are unknown and evidence is lacking regarding whether poor movement behavior temporally precedes altered lipid levels (7, 10, 11). Clarifying potential temporal associations of objectively measured movement behavior with lipid indices is an important inquiry that has implications for mounting effective childhood dyslipidemia prevention programs (6-8, 10). Moreover, wearable devices are becoming important in prevention, early detection, screening, and disease management (12).

The present study (1) examined the longitudinal associations of cumulative accelerometer-measured sedentary time, LPA, and MVPA, with repeated measures of fasting plasma high-density lipoprotein cholesterol, low-density lipoprotein cholesterol, triglyceride, and total cholesterol in 11-year-old children followed-up for 13 years; (2) assessed the extent to which the associations of movement behavior with lipid indices is mediated by fat mass, lean mass, insulin resistance, and inflammation; and (3) examined the temporal interrelations of movement behaviors with lipid indices using data from the Avon Longitudinal Study of Parents and Children (ALSPAC) birth cohort, England. It was hypothesized that increased sedentary time and decreased PA would worsen the lipid profile which could be mediated by increased adiposity.

## Materials and Methods

### Study Cohort

Data were from the ALSPAC birth cohort, which investigates factors that influence childhood development and growth. Pregnant women resident in Avon, UK, with expected dates of delivery between April 1 1991 and December 31 1992 were invited to take part in the study. A total of 20 248 pregnancies were identified as being eligible and the initial number of pregnancies enrolled was 14 541. Of the initial pregnancies, there was a total of 14 676 fetuses, resulting in 14 062 live births and 13 988 children who were alive at 1 year of age. When the oldest children were approximately 7 years of age, an attempt was made to bolster the initial sample with eligible cases who had failed to join the study originally. As a result, when considering variables collected from the age of 7 onwards (and potentially abstracted from obstetric notes) there are data available for more than the 14 541 pregnancies mentioned above. The number of new pregnancies not in the initial sample (known as Phase I enrollment) that are currently represented in the released data and reflecting enrollment status at the age of 24 is 906, resulting in an additional 913 children being enrolled (456, 262, and 195 recruited during Phases II, III, and IV respectively). The total sample size for analyses using any data collected after the age of 7 was therefore 15 447 pregnancies, resulting in 15 658 fetuses. Of these 14 901 children were alive at 1 year of age. Regular clinic visits of the children commenced at 7 years of age and are still ongoing into adulthood. Study data at 24 years of age were collected and managed using REDCap electronic data capture tools (13). In this study, of 2040 participants with at least 2 time-points of valid sedentary time, LPA, and MVPA measurements at either age 11, 15, or 24 years clinic visit only 792 participants with complete high-density lipoprotein cholesterol, low-density lipoprotein cholesterol, triglyceride, and total cholesterol measures at 15, 17, and 24 years clinic visits were eligible for analyses (Fig. S1 (14)). The excluded participants who had 1 or no time-point measure of sedentary time and PA during the 13-year-long follow-up study had similar baseline characteristics with those included in the study (Table S1 (14)). Ethics approval for the study was obtained from the ALSPAC Ethics and Law Committee and the Local Research Ethics Committees. Informed consent for the use of data collected via questionnaires and clinics was obtained from participants following the recommendations of the ALSPAC Ethics and Law Committee at the time (15-17). Consent for biological samples has been collected in accordance with the Human Tissue Act (2004). Please note that the study website contains details of all the data that are available through a fully searchable data dictionary and variable search tool (http://www.bristol.ac.uk/alspac/researchers/our-data/).

### Sedentary Time and Physical Activity Assessment

Sedentary time, LPA, and MVPA were assessed with an ActiGraphTM (LLC, Fort Walton Beach, FL, USA) accelerometer worn on the waist for 7 consecutive days at the 11- and 15-year clinic visits whereas movement behavior at 24 year was assessed using an ActiGraph GT3X+ accelerometer device worn for 4 consecutive days (11, 18). Participants were instructed to wear the device from first thing in the morning until they went to bed. A valid day was defined as providing data for at least 10 hours per day (excluding sequences of 10 or more minutes with consecutive 0 counts) and children were only included in the analyses if they provided at least 3 valid days of recording. The devices capture movement in terms of acceleration as a combined function of frequency and intensity. Data are recorded as counts that result from summing postfiltered accelerometer values (raw data at 30 Hz) into 60-second epoch units. Data were processed using Kinesoft software, version 3.3.75 (Kinesoft), according to an established protocol (19). Activity counts per minute threshold validated in young people were used to calculate the amount of time spent: MVPA, >2296 counts per minute (cpm); LPA, 100 to 2296 cpm; and sedentary time, 0 to <100 cpm at ages 11 and 15 years using the Evenson cutpoint whereas, at the 24-year assessment, the 2020 cpm Troiano cut point was used (3, 19, 20). The Evenson cut point used in stratifying activity threshold has shown the best overall performance across all intensity levels and was suggested to be the most appropriate cut point for youth (21, 22).

### Lipid Assessments

There were no measures of fasting plasma lipids at age 11 years. Fasting plasma high-density lipoprotein cholesterol, low-density lipoprotein cholesterol, triglyceride, and total cholesterol were assessed at the 15-, 17-, and 24-year clinic visits, and a detailed assessment has been reported (coefficient of variation was <5%) (20, 23-25). Using standard protocols, fasting plasma samples at ages 15, 17, and 24 years were collected, spun, and frozen at −80 °C.

### Anthropometry and Body Composition

Anthropometry (height and weight) at ages 11, 15, and 24 years were assessed in line with standard protocols, and body mass index was computed as weight in kilograms per height in meters squared (20, 23). Body composition (total fat mass and lean mass) was assessed using a dual-energy X-ray absorptiometry scanner at 11, 15, and 24 years clinic visits as previously described (20, 23, 24).

### Cardiometabolic, Socioeconomic, and Lifestyle Factors

Heart rate and systolic and diastolic blood pressure were measured with an Omron monitor at ages 11, 15, and 24 years as previously detailed (20, 23). A detailed assessment of fasting high-sensitivity C-reactive protein and glucose has been described (20, 23, 24). Fasting insulin was assessed using an ultrasensitive automated microparticle enzyme immunoassay (Mercodia), which does not cross-react with proinsulin; the sensitivity of the immunoassay was 0.07 mU/L (26). The homeostatic model assessment of insulin resistance was calculated by fasting plasma insulin × fasting plasma glucose/22.5 (27, 28). At the 17-year clinic visit, participants were briefly asked about their personal and family (mother, father, and siblings) medical history such as a history of hypertension, diabetes, high cholesterol, and vascular disease. All participants had attained puberty at the 17-year clinic visit using a time (years) to age at peak height velocity objective assessment derived using superimposition by translation and rotation mixed-effects growth curve analysis (3, 20, 29). The socioeconomic status of the participant's mother was grouped according to the 1991 British Office of Population and Census Statistics classification (30). Questionnaires to assess smoking behavior were administered at the 13-, 15-, and 24-year clinic visits. A specific question regarding whether participants smoked in the last 30 days was used as an indicator of current smoking status.

### Statistical Analysis

Cohort descriptive characteristics were summarized as means and SD, medians, and interquartile ranges, or frequencies and percentages. Sex differences were explored using independent t-tests, Mann–Whitney U tests, or chi-square tests for normally distributed, skewed, or dichotomous variables, respectively. Multicategory variables were analyzed using one-way analysis of variance. Normality was assessed by histogram curve, quantile–quantile plot, and Kolmogorov–Smirnov tests. Logarithmic transformation of skewed variables was conducted and normality was confirmed prior to further analysis.

#### Mediation path analyses

Mediating path analyses using structural equation models separately examined the mediating role of cumulative total fat mass and lean mass on the longitudinal associations of cumulative sedentary time, LPA, or MVPA with each of cumulative high-density lipoprotein cholesterol, low-density lipoprotein cholesterol, triglyceride, and total cholesterol. Analyses were adjusted for age, sex, insulin resistance, high-sensitivity C-reactive protein, family history of hypertension and cardiovascular diseases, smoking status, socioeconomic status, heart rate, total fat mass, lean mass, sedentary time, LPA, MVPA, and high-density lipoprotein cholesterol, low-density lipoprotein cholesterol, triglyceride, or total cholesterol depending on the mediator, predictor, or outcome. The path models had 3 equations per regression analysis: the longitudinal associations of cumulative sedentary time, LPA, or MVPA with cumulative total fat mass, lean mass, insulin resistance, or inflammation (Equation 1); the longitudinal associations of cumulative total fat mass or lean mass with high-density lipoprotein cholesterol, low-density lipoprotein cholesterol, triglyceride, and total cholesterol (Equation 2); and the longitudinal associations of cumulative sedentary time, LPA, and MVPA with cumulative high-density lipoprotein cholesterol, low-density lipoprotein cholesterol, triglyceride and total cholesterol (Equation 3, total effect), and Equation 3′ (direct effect) accounted for the mediating role of total fat mass, lean mass, insulin resistance or inflammation on the longitudinal associations of cumulative sedentary time, LPA, and MVPA with cumulative high-density lipoprotein cholesterol, low-density lipoprotein cholesterol, triglyceride, and total cholesterol. The proportion of mediating or suppressing roles was estimated as the ratio of the difference between Equation 3 and Equation 3′ or the multiplication of Equations 1 and 2 divided by Equation 3 and expressed as a percentage. A mediating or indirect role is confirmed when there are statistically significant associations between (1) the predictor and mediator, (2) the predictor and outcome, (3) the mediator and outcome, and when (4) the longitudinal associations between the predictor and outcome variable was attenuated upon inclusion of the mediator (31). However, when the magnitude of the longitudinal association between the predictor and outcome is increased upon inclusion of a third variable, a suppression is confirmed (31). Path analyses were conducted with 1000 bootstrapped samples.

#### Temporal path analyses

Lastly, we used structural equation modeling with an autoregressive cross-lagged design to examine the separate temporal associations of sedentary time, LPA, and MVPA with each of high-density lipoprotein cholesterol, low-density lipoprotein cholesterol, triglyceride, and total cholesterol at ages 15 and 24 years only, due to the lack of fasting lipid data at age 11 years. The cross-lagged models first tested the separate associations of sedentary time, LPA, and MVPA at 15 years with each of high-density lipoprotein cholesterol, low-density lipoprotein cholesterol, triglyceride, and total cholesterol at 24 years. Next, the separate associations of high-density lipoprotein cholesterol, low-density lipoprotein cholesterol, triglyceride, and total cholesterol at 15 years with sedentary time, LPA, and MVPA at 24 years were examined. These models were adjusted for all covariates measured at 15 years. In the cross-lagged design, the potential association could be sedentary time, LPA, and MVPA leading to lipid levels, lipid levels leading to sedentary time, LPA, and MVPA, or bidirectional associations of sedentary time, LPA, and MVPA with lipid levels. If a path from sedentary time, LPA, and MVPA at time t-1 (15 years) to each of high-density lipoprotein cholesterol, low-density lipoprotein cholesterol, and triglyceride at time t-2 (24 years) reached significance (*P* < .05), changes in the earlier variables are considered to lead to changes in the later, and vice versa. A stronger predictive effect is determined by a larger standardized regression coefficient. We concluded that the cross-lagged models had good fits with the following indices: the root mean square error of approximation <0.05, the normed fit index, relative fit index, incremental fit index, Tucker–Lewis fit index, and comparative fit index, >0.90 for all (32). Error terms were included in the cross-lagged model.

Collinearity diagnoses were performed and accepted results with a variance inflation factor <5, considered differences and associations with a 2-sided *P* < .05 to be statistically significant, and made conclusions based on effect estimates and their CIs. Covariates were identified based on previous studies (7, 8, 10, 20, 24, 30, 33-35). Analyses involving 800 ALSPAC children at 0.8 statistical power, 0.05 alpha, and 2-sided *P* value would show a minimum detectable effect size of 0.09 SD if they had relevant exposure for a normally distributed quantitative variable (36). All statistical analyses were performed using SPSS statistics software, Version 27.0 (IBM Corp, Armonk, NY, USA), and mediation analyses and autoregressive cross-lagged temporal path structural equation modeling was conducted using IBM AMOS version 27.0.

## Results

**Figure 1.**
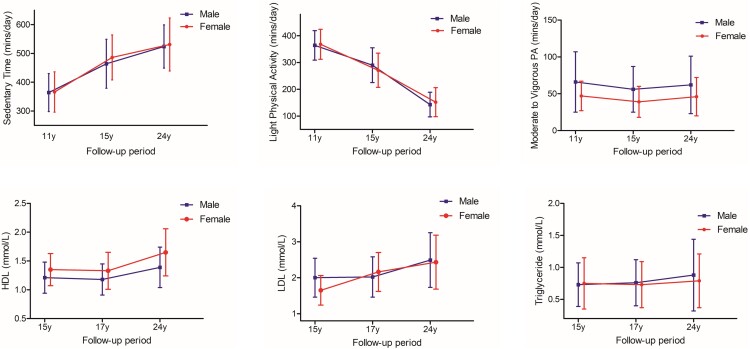
Characteristics of movement behavior and lipid profile of participants from childhood through young adulthood (Males n = 337, Females n = 455). Sedentary time, light physical activity, moderate to vigorous physical activity, HDL and LDL are presented as means and SD. Triglycerides are presented as median and interquartile range. HDL, high-density lipoprotein cholesterol; LDL, low-density lipoprotein cholesterol.

Altogether 792 participants who had at least 2 time-point measures of sedentary time, LPA, and MVPA during the 11, 15, and 24-year clinic visits with complete fasting high-density lipoprotein cholesterol, low-density lipoprotein cholesterol, triglyceride, and total cholesterol measures at ages 15, 17, and 24 years were included (Fig. S1 (14)). The excluded participants who had 1 or no time-point measure of movement behavior during the 13-year-long follow-up study had characteristics similar to those included in the study (Table S1 (14)). Sedentary time increased, LPA decreased, and MVPA was U-shaped from ages 11 through 24 years in both males and females (Table 1 and Fig. 1). From ages 15 to 24 years, lipid indices increased (Table 1 and Fig. 1). Other characteristics are described in Table 1.

**Table 1.** Descriptive characteristics of cohort participants

	11 years				15 years				24 years		
Age at clinic visits/follow-up Variables	Male (n = 337)	Female (n = 455)	*P* value		Male (n = 337)	Female (n = 455)	*P* value		Male (n = 337)	Female (n = 455)	*P* value
**Anthropometry**											
Age at clinic visit (years)	11.70 (0.20)	11.71 (0.21)	.738		15.38 (0.22)	15.39 (0.24)	.634		24.57 (0.79)	24.45 (0.77)	.033
Height (m)	1.51 (0.07)	1.52 (0.07)	.139		1.75 (0.07)	1.65 (0.06)	<.0001		1.80 (0.07)	1.67 (0.06)	<.0001
*^a^*Weight (kg)	41.20 (11.9)	42.0 (12.0)	.208		63.0 (13.1)	56.80 (10.9)	<.0001		79.30 (17.32)	64.15 (16.55)	<.0001
Ethnicity, White (n,%)	303 (96.2)	410 (97.9)	.188		NA				NA		
**Body composition**											
*^a^*Total fat mass (kg)	8.41 (8.40)	10.76 (7.52)	<.0001		8.59 (7.51)	16.80 (8.79)	<.0001		18.53 (11.60)	21.16 (11.12)	<.0001
*^a^*Lean mass (kg)	29.86 (5.29)	28.69 (6.39)	<.0001		50.0 (8.59)	36.89 (4.81)	<.0001		56.90 (10.99)	40.93 (6.34)	<.0001
*^a^*Body mass index (kg/m^2^)	18.07 (4.27)	18.15 (3.95)	.579		20.31 (3.73)	20.83 (4.08)	.009		24.38 (5.10)	23.09 (5.57)	<.0001
**Vascular measures**											
Heart rate (beat/mins)	74 (11)	78 (11)	<.0001		72 (12)	77 (11)	<.0001		65 (11)	68 (9)	<.0001
Systolic blood pressure (mmHg)	105 (9)	105 (10)	.891		127 (10)	120 (10)	<.0001		123 (10)	111 (9)	<.0001
Diastolic blood pressure (mmHg)	58 (6)	58 (6)	.919		68 (8)	66 (9)	.012		67 (8)	66 (8)	.009
**Lifestyle factors**											
Smoked in the last 30 days (n,%)	<5 (0.3)	8 (1.8)	.086		24 (7.3)	55 (12.2)	.030		82 (24.6)	105 (23.2)	.672
Family history of H-D-C-V (n,%)	94 (31.6)	118 (28.1)	.319		NA				NA		
Sedentary time (min/day)	364 (66)	366 (70)	.807		464 (85)	486 (78)	.001		524 (75)	531 (92)	.615
Light physical activity (min/day)	364 (55)	368 (56)	.317		290 (65)	271 (64)	<.0001		143 (46)	152 (54)	.246
MVPA (min/day)	66 (41)	47 (20)	<.0001		56 (31)	39 (21)	<.0001		62 (39)	46 (26)	.001
**Maternal social economic status (n, %)**			.230		NA				NA		
Professional	17 (9.9)	13 (6.0)									
Managerial and technical	74 (43)	83 (38.4)									
Skilled non-manual	49 (28.5)	76 (35.2)									
Skilled manual	<8 (1.2)	<8 (2.3)									
Partly skilled	25 (14.5)	31 (14.4)									
Unskilled	<8 (2.9)	8 (3.7)									
**Fasting plasma metabolic indices**	**15 years**				**17 years**				**24 years**		
Total cholesterol (mmol/L)	3.57 (0.60)	3.90 (0.62)	<.0001		3.58 (0.60)	3.91 (0.69)	<.0001		4.36 (0.82)	4.49 (0.83)	.028
High-density lipoprotein (mmol/L)	1.21 (0.27)	1.35 (0.28)	<.0001		1.18 (0.27)	1.33 (0.32)	<.0001		1.39 (0.35)	1.65 (0.41)	<.0001
Low-density lipoprotein (mmol/L)	2.00 (0.54)	1.65 (0.41)	<.0001		2.02 (0.56)	2.16 (0.54)	<.0001		2.49 (0.76)	2.43 (0.75)	.310
*^a^*Triglyceride (mmol/L)	0.73 (0.34)	0.75 (0.40)	.106		0.76 (0.36)	0.73 (0.36)	.489		0.88 (0.56)	0.79 (0.42)	<.0001
Glucose (mmol/L)	5.30 (0.36)	5.14 (0.34)	<.0001		5.14 (0.36)	4.90 (0.33)	<.0001		5.49 (1.03)	5.21 (0.52)	<.0001
*^a^*Insulin (mU/L)	8.09 (4.78)	9.74 (5.54)	<.0001		5.80 (4.31)	7.51 (4.14)	<.0001		7.38 (5.32)	7.68 (5.93)	.413
*^a^*High sensitivity C-reactive protein (mg/L)	0.34 (0.60)	0.34 (0.59)	.240		0.45 (0.59)	0.60 (1.30)	.005		0.64 (1.41)	1.00 (1.95)	<.0001

The values are means (SD) unless stated otherwise.

Abbreviations: H-D-C-V, hypertension, diabetes, high cholesterol, and vascular disease; MVPA, moderate to vigorous physical activity; NA, not available/applicable; *P* value for sex differences.

^*a*^Median (interquartile range) except for lifestyle factors and ethnicity. Differences between sexes were tested using Student's t-test for normally distributed continuous variables, Mann–Whitney U test for skewed continuous variables, Chi-square test for dichotomous variables, and analysis of covariance for the multicategory variable. The denominators used for calculating the percentages may vary depending on data availability at each study time point. A 2-sided *P* < .05 is considered to be statistically significant.

### Mediating or Suppressing Effects of Body Composition, Insulin Resistance, and Inflammation in the Associations of Sedentary Time, LPA, and MVPA With Lipid Indices

Cumulative sedentary time was positively associated with cumulative high-density lipoprotein cholesterol, low-density lipoprotein cholesterol, triglyceride, and total cholesterol (Table 2). Insulin resistance partly mediated the positive associations of sedentary time with high-density lipoprotein cholesterol, but suppressed the associations with low-density lipoprotein cholesterol, triglyceride, and total cholesterol (Table 2). Lean mass partly suppressed the positive associations of sedentary time with high-density lipoprotein cholesterol and mediated the associations with triglyceride (Table 2). Fat mass and inflammation had no significant mediating effect on the associations between sedentary time and lipid indices.

**Table 2.** Mediating or suppressing role of cumulative fasting metabolic indices and inflammation on the longitudinal associations of cumulative sedentary time with cumulative lipids

Cumulative sedentary time from ages 11 to 24 years N = 792							
	Total effect		Direct effect		Indirect effect		Mediation or suppression (%)
Mediators	β (95% CI)	*P* value	β (95% CI)	*P* value	β (95% CI)	*P* value	
**Cumulative fasting high-density lipoprotein cholesterol from ages 15 to 24 years**							
Total fat mass	0.303 (0.259-0.345)	.**002**	0.303 (0.259-0.344)	.**002**	0.000 (−0.005-0.003)	.838	0
Lean mass	0.376 (0.332-0.420)	.**002**	0.394 (0.348-0.439)	.**002**	−0.017 (−0.027-−0.008)	.**003**	**4.5** suppression
Insulin resistance	0.290 (0.253-0.332)	.**002**	0.268 (0.229-0.309)	.**002**	0.022 (0.014-0.035)	.**001**	**7.6** mediation
High-sensitivity CRP	0.305 (0.265-0.347)	.**002**	0.308 (0.267-0.348)	.**002**	−0.003 (−0.014-0.007)	.482	1.0
**Cumulative fasting low-density lipoprotein cholesterol from ages 15 to 24 years**							
Total fat mass	0.060 (0.018-0.101)	.**003**	0.059 (0.018-0.098)	.**003**	0.001 (−0.011-0.016)	.796	1.7
Lean mass	0.148 (0.103-0.187)	.**003**	0.144 (0.097-0.186)	.**002**	0.003 (−0.001-0.099)	.166	2.1
Insulin resistance	0.146 (0.107-0.184)	.**002**	0.178 (0.142-0.215)	.**002**	−0.032 (−0.048-−0.019)	.**001**	**21.9** suppression
High-sensitivity CRP	0.136 (0.096-0.174)	.**002**	0.131 (0.091-0.168)	.**002**	0.006 (−0.006-0.017)	.287	4.4
**Cumulative fasting triglyceride from ages 15 to 24 years**							
Total fat mass	0.020 (−0.022-0.064)	.353	0.019 (−0.024-0.058)	.434	0.002 (−0.011-0.016)	.758	10.0
Lean mass	0.046 (0.001-0.085)	.**042**	0.031 (−0.016-0.072)	.253	0.015 (0.008-0.025)	.**002**	**32.6** mediation
Insulin resistance	0.096 (0.052-0.132)	.**004**	0.148 (0.111-0.180)	.**003**	−0.052 (−0.078-−0.030)	.**001**	**54.2** suppression
High-sensitivity CRP	0.084 (0.042-0.121)	.**004**	0.075 (0.032-0.110)	.**004**	0.009 (−0.007-0.024)	.242	10.7
**Cumulative fasting total cholesterol from ages 15 to 24 years**							
Total fat mass	0.192 (0.153-0.235)	.**002**	0.192 (0.153-0.231)	.**002**	0.000 (−0.013-0.015)	.969	0
Lean mass	0.309 (0.264-0.346)	.**003**	0.311 (0.265-0.352)	.**003**	−0.002 (−0.007-0.002)	.284	0.65
Insulin resistance	0.278 (0.238-0.312)	.**002**	0.313 (0.279-0.347)	.**002**	−0.036 (−0.053-−0.023)	.**001**	**13.0** suppression
High-sensitivity CRP	0.276 (0.240-0.313)	.**002**	0.274 (0.238-0.311)	.**002**	0.002 (−0.006-0.011)	.516	0.73

Mediation structural equation model was adjusted for sex, family history of hypertension/diabetes/high cholesterol/vascular disease, socioeconomic status, and time-varying covariates measured at both baseline and follow-up, such as age, high sensitivity C-reactive protein (CRP), heart rate, systolic blood pressure, smoking status, and fat mass, lean mass, insulin resistance, light physical activity, and moderate to vigorous physical activity, with additional adjustments for high-density lipoprotein cholesterol, low-density lipoprotein cholesterol, or triglyceride depending on the mediator and outcome. β is the standardized regression coefficient. *P* < .05 was considered to be statistically significant and are in bold. Total cholesterol analyses was not adjusted for lipids. When the magnitude of the association between exposure and outcome is increased upon inclusion of a third variable, suppression occurred but was mediated if decreased.

**Figure 2.**
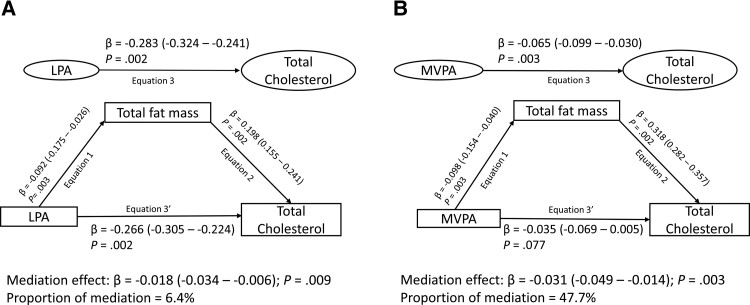
Mediating effect of increased total fat mass on the longitudinal associations of light physical activity (A) and moderate to vigorous physical activity (B) from childhood with total cholesterol. Mediation structural equation model was adjusted for sex, family history of hypertension/diabetes/high cholesterol/vascular disease, socioeconomic status, and time-varying covariates measured at both baseline and follow-up such as age, high sensitivity C-reactive protein, heart rate, systolic blood pressure, smoking status, lean mass, insulin resistance, sedentary time, and light physical activity or moderate to vigorous physical activity depending on the predictor. β is the standardized regression coefficient. *P* < .05 was considered to be statistically significant.

Cumulative LPA was inversely associated with cumulative high-density lipoprotein cholesterol, low-density lipoprotein cholesterol, and triglyceride (Table 3). Total fat mass partly suppressed the inverse associations of LPA with high-density lipoprotein cholesterol, but partly mediated the associations with low-density lipoprotein cholesterol, triglyceride, and total cholesterol (Fig. 2 and Table 3). Lean mass, insulin resistance, and inflammation had no significant mediating effect on the associations between LPA and lipid indices.

**Table 3.** Mediating or suppressing role of cumulative fasting metabolic indices and inflammation on the longitudinal associations of light physical activity with cumulative lipids

Cumulative light physical activity from ages 11 to 24 years N = *792*							
	Total effect		Direct effect		Indirect effect		Mediation or Suppression (%)
Mediators	β (95% CI)	*P* value	β (95% CI)	*P* value	β (95% CI)	*P* value	
**Cumulative fasting high-density lipoprotein cholesterol from ages 15 to 24 years**							
Total fat mass	−0.333 (−0.371-−0.290)	.**003**	−0.341 (−0.384-−0.297)	.**003**	0.008 (0.002-0.020)	.**010**	**2.4** suppression
Lean mass	−0.459 (−0.502-−0.413)	.**002**	−0.456 (−0.498-−0.407)	.**002**	−0.004 (−0.018-0.011)	.642	0.9
Insulin resistance	−0.295 (−0.332-−0.254)	.**003**	−0.291 (−0.328-−0.254)	.**003**	−0.005 (−0.020-0.010)	.513	1.7
High-sensitivity CRP	−0.335 (−0.373-−0.293)	.**003**	−0.332 (−0.371-−0.292)	.**002**	−0.003 (−0.017-0.012)	.649	0.9
**Cumulative fasting low-density lipoprotein cholesterol from ages 15 to 24 years**							
Total fat mass	−0.141 (−0.187-−0.095)	.**002**	−0.123 (−0.166-−0.078)	.**002**	−0.018 (−0.036-−0.005)	.**012**	**12.8** mediation
Lean mass	−0.242 (−0.291-−0.197)	.**001**	−0.241 (−0.288-−0.195)	.**001**	−0.001 (−0.005-0.001)	.315	0.4
Insulin resistance	−0.223 (−0.266-−0.184)	.**002**	−0.229 (−0.266-−0.192)	.**002**	0.006 (−0.013-0.026)	.518	2.7
High-sensitivity CRP	−0.187 (−0.227-−0.144)	.**002**	−0.191 (−0.229-−0.149)	.**002**	0.003 (−0.009-0.016)	.610	1.6
**Cumulative fasting triglyceride from ages 15 to 24 years**							
Total fat mass	−0.067 (−0.110-−0.023)	.**002**	−0.048 (−0.094-−0.004)	.**030**	−0.019 (−0.039-−0.005)	.**017**	**28.4** mediation
Lean mass	−0.071 (−0.117-−0.019)	.**008**	−0.073 (−0.120-−0.022)	.**005**	0.002 (−0.005-0.011)	.520	2.8
Insulin resistance	−0.158 (−0.204-−0.104)	.**002**	−0.169 (−0.204-−0.131)	.**002**	0.011 (−0.021-0.047)	.468	7.0
High-sensitivity CRP	−0.101 (−0.146-−0.057)	.**002**	−0.107 (−0.146-−0.063)	.**003**	0.005 (−0.015-0.023)	.632	5.0
**Cumulative fasting total cholesterol from ages 15 to 24 years**							
Total fat mass	−0.283 (−0.324-−0.241)	.**002**	−0.266 (−0.305-−0.224)	.**002**	−0.018 (−0.034-−0.006)	.**009**	**6.4** mediation
Lean mass	−0.427 (−0.474-−0.385)	.**001**	−0.426 (−0.472-−0.384)	.**001**	−0.002 (−0.008-0.005)	.567	0.47
Insulin resistance	−0.363 (−0.402-−0.325)	.**002**	−0.368 (−0.401-−0.332)	.**002**	0.005 (−0.013-0.024)	.605	1.4
High-sensitivity CRP	−0.337 (−0.372-−0.297)	.**002**	−0.339 (−0.373-−0.300)	.**003**	0.002 (−0.007-0.012)	.706	0.59

Mediation structural equation model was adjusted for sex, family history of hypertension/diabetes/high cholesterol/vascular disease, socioeconomic status, and time-varying covariates measured at both baseline and follow-up such as age, high sensitivity C-reactive protein (CRP), heart rate, systolic blood pressure, smoking status, and fat mass, lean mass, insulin resistance, sedentary time, and moderate-to-vigorous physical activity, with additional adjustments for high-density lipoprotein cholesterol, low-density lipoprotein cholesterol, or triglyceride depending on the mediator and outcome. β is the standardized regression coefficient. *P* < .05 was considered to be statistically significant and are in bold. Total cholesterol analyses was not adjusted for lipids. When the magnitude of the association between the exposure and the outcome is increased upon inclusion of a third variable, suppression occurred but if decreased mediation occurred.

**Figure 3.**
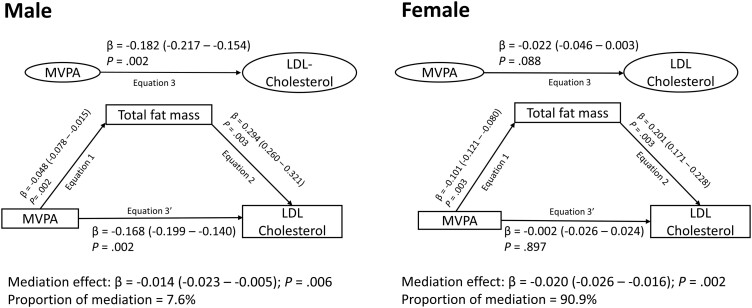
Mediating effect of increased total fat mass on the longitudinal associations of moderate to vigorous physical activity from childhood with low-density lipoprotein cholesterol in males and females . The mediation structural equation model was adjusted for family history of hypertension/diabetes/high cholesterol/vascular disease, socioeconomic status, and time-varying covariates measured at both baseline and follow-up, such as age, high sensitivity C-reactive protein, triglyceride, high-density lipoprotein cholesterol, heart rate, systolic blood pressure, smoking status, lean mass, insulin resistance, sedentary time, and light physical activity. β is the standardized regression coefficient. *P* < .05 was considered to be statistically significant.

Cumulative MVPA was inversely associated with cumulative high-density lipoprotein cholesterol, low-density lipoprotein cholesterol, and triglyceride (Table 4). Fat mass had a 37% and 48% mediation effect on the inverse associations of MVPA with low-density lipoprotein cholesterol and total cholesterol, respectively, but not with high-density lipoprotein cholesterol and triglyceride (Fig. 2 and Table 4). Fat mass had an 8% and 91% mediation effect on the inverse associations of MVPA with low-density lipoprotein cholesterol in males and females, respectively (Fig. 3), but no significant differences with other lipid indices (data not shown). Lean mass partially suppressed the inverse associations of MVPA with low-density lipoprotein cholesterol, triglyceride, and total cholesterol (Table 4). Insulin resistance and inflammation had no significant mediating or suppressing effect on the associations between MVPA and lipid indices.

**Table 4.** Mediating or suppressing role of cumulative fasting metabolic indices and inflammation on the longitudinal associations of cumulative moderate to vigorous physical activity with cumulative lipids

Cumulative moderate to vigorous physical activity from ages 11 to 24 years (N *=* 792*)*							
	Total effect		Direct effect		Indirect effect		Mediation or suppression (%)
Mediators	β (95% CI)	*P* value	β (95% CI)	*P* value	β (95% CI)	*P* value	
**Cumulative fasting high-density lipoprotein cholesterol from ages 15 to 24 years**							
Total fat mass	−0.027 (−0.063-0.008)	.113	−0.019 (−0.056-0.015)	.252	−0.007 (−0.014-−0.003)	.002	25.9
Lean mass	−0.040 (−0.077-−0.006)	.**016**	−0.041 (−0.077-−0.007)	.**016**	0.001 (−0.001-0.003)	.515	2.5
Insulin resistance	−0.054 (−0.092-−0.020)	.**006**	−0.052 (−0.089-−0.018)	.**007**	−0.002 (−0.010-0.005)	.478	3.7
High-sensitivity CRP	−0.048 (−0.084-−0.013)	.**013**	−0.049 (−0.084-−0.013)	.**014**	0.001 (−0.002-0.004)	.583	2.1
**Cumulative fasting low-density lipoprotein cholesterol from ages 15 to 24 years**							
Total fat mass	−0.063 (−0.096-−0.022)	.**003**	−0.039 (−0.074-0.005)	.091	−0.023 (−0.038-−0.010)	.**003**	**36.5** mediation
Lean mass	−0.107 (−0.142-−0.068)	.**002**	−0.113 (−0.148-−0.073)	.**003**	0.007 (0.003-0.011)	.**003**	**6.5** suppression
Insulin resistance	−0.091 (−0.127-−0.052)	.**002**	−0.094 (−0.129-−0.054	.**002**	0.002 (−0.005-0.011)	.510	2.2
High-sensitivity CRP	−0.089 (−0.123-−0.052)	.**002**	−0.089 (−0.124-−0.050)	.**002**	−0.001 (−0.007-0.006)	.754	1.1
**Cumulative fasting triglyceride from ages 15 to 24 years**							
Total fat mass	−0.012 (−0.045-0.024)	.547	0.011 (−0.021-0.053)	.472	−0.023 (−0.037-−0.010)	.004	191.6
Lean mass	−0.056 (−0.087-−0.018)	.**003**	−0.065 (−0.100-−0.028)	.**003**	0.010 (0.005-0.016)	.**004**	**17.9** suppression
Insulin resistance	−0.027 (−0.057-0.009)	.147	−0.032 (−0.065-0.001)	.061	0.005 (−0.009-0.022)	.441	18.5
High-sensitivity CRP	−0.031 (−0.064-0.008)	.109	−0.030 (−0.063-0.008)	.131	−0.001 (−0.010-0.008)	.791	3.2
**Cumulative fasting total cholesterol from ages 15 to 24 years**							
Total fat mass	−0.065 (−0.099-−0.030)	.**003**	−0.035 (−0.069-0.005)	.077	−0.031 (−0.049-−0.014)	.**003**	**47.7** mediation
Lean mass	−0.121 (−0.154-−0.085)	.**003**	−0.129 (−0.163-−0.094)	.**003**	0.008 (0.004-0.014)	.**003**	**6.6** suppression
Insulin resistance	−0.108 (−0.141-−0.074)	.**003**	−0.109 (−0.142-−0.073)	.**003**	0.000 (−0.007-0.008)	.924	0
High-sensitivity CRP	−0.105 (−0.136-−0.069)	.**004**	−0.162 (−0.135-−0.067)	.**003**	−0.002 (−0.008-0.005)	.470	1.9

The mediation structural equation model was adjusted for sex, family history of hypertension/diabetes/high cholesterol/vascular disease, socioeconomic status, and time-varying covariates measured at both baseline and follow-up, such as age, high sensitivity C-reactive protein (CRP), heart rate, systolic blood pressure, smoking status, and fat mass, lean mass, insulin resistance, sedentary time, and light physical activity, with additional adjustments for high-density lipoprotein cholesterol, low-density lipoprotein cholesterol, or triglyceride depending on the mediator and outcome. β is standardized regression coefficient. *P* < .05 was considered to be statistically significant and are in bold. Total cholesterol analyses was not adjusted for lipids. When the magnitude of the association between the exposure and outcome is increased upon inclusion of a third variable suppression occurred but mediation, if decreased. Whenever the total effect is not statistically significant a subsequent significant indirect effect is not bolded due to the criteria for defining mediators.

### Temporal (Cross-lagged) and Inter-relational (Autoregressive) Associations of Sedentary Time, LPA, and MVPA With Lipid Indices

MVPA, high-density lipoprotein cholesterol, low-density lipoprotein cholesterol, triglyceride, and total cholesterol at age 15 years were positively associated with their individual variables at age 24 years; however, sedentary time and LPA at age 15 years was inversely associated with their individual variables at age 24 years (Table 5). Higher MVPA at 15 years was associated with lower low-density lipoprotein cholesterol at 24 years but low-density lipoprotein cholesterol at 15 years was not associated with MVPA at 24 years (Table 5). There were no temporal or bidirectional relationships of sedentary time, LPA, or MVPA with high-density lipoprotein cholesterol, triglyceride, or total cholesterol. Sedentary time or LPA had no temporal relations with low-density lipoprotein cholesterol or total cholesterol (Table 5).

**Table 5.** Autoregressive cross-lagged temporal causal longitudinal analyses of sedentary time and physical activity with lipids at 15 and 24 years of age

N = 792 participants										
High-density lipoprotein cholesterol						Low-density lipoprotein cholesterol				
Autoregressive	B	β	SE	*P* value		Autoregressive	B	β	SE	*P* value
ST T1 → ST T2	−0.372	−0.348	0.179	.**038**		ST T1 → ST T2	−0.423	−0.397	0.177	.**017**
LPA T1 → LPA T2	−0.040	−0.052	0.165	.806		LPA T1 → LPA T2	−0.067	−0.086	0.164	**<**.**0001**
MVPA T1 → MVPA T2	0.814	0.684	0.142	**<**.**0001**		MVPA T1 → MVPA T2	0.822	0.689	0.139	**<**.**0001**
HDL T1 → HDL T2	0.775	0.588	0.039	**<**.**0001**		LDL T1 → LDL T2	0.684	0.555	0.040	**<**.**0001**
**Cross-lagged**										
ST T1 → HDL T2	0.000	0.024	0.000	.504		ST T1 → LDL T2	0.000	0.047	0.000	.218
HDL T1 → ST T2	−9.559	−0.033	23.597	.685		LDL T1 → ST T2	3.029	0.021	11.703	.796
LPA T1 → HDL T2	0.000	0.048	0.000	.168		LPA T1 → LDL T2	0.000	0.002	0.000	.967
HDL T1 → LPA T2	3.324	0.020	14.286	.816		LDL T1 → LPA T2	5.178	0.062	6.916	.454
MVPA T1 → HDL T2	0.000	0.031	0.000	.359		MVPA T1 → LDL T2	−0.002	−0.082	0.001	.**022**
HDL T1 → MVPA T2	1.815	0.017	8.667	.834		LDL T1 → MVPA T2	1.493	0.028	4.256	.726
**Triglyceride**						**Total cholesterol**				
ST T1 → ST T2	−0.385	−0.360	0.179	.**032**		ST T1 → ST T2	−0.440	−0.415	0.176	.**012**
LPA T1 → LPA T2	−0.053	−0.067	0.164	.749		LPA T1 → LPA T2	−0.011	−0.014	0.166	.946
MVPA T1 → MVPA T2	0.823	0.688	0.139	**<**.**0001**		MVPA T1 → MVPA T2	0.820	0.687	0.130	**<**.**0001**
Triglyceride T1 → Triglyceride T2	0.427	0.361	0.042	**<**.**0001**		Total cholesterol T1 → Total cholesterol T2	0.731	0.562	0.041	**<**.**0001**
**Cross-lagged**						**Cross-lagged**				
ST T1 → Triglyceride T2	0.000	0.026	0.000	.528		ST T1 → Total cholesterol T2	0.000	0.048	0.000	.205
Triglyceride T1 → ST T2	−9.117	−0.016	46.041	.843		Total cholesterol T1 → ST T2	−7.699	−0.056	10.816	.843
LPA T1 → Triglyceride T2	0.000	−0.054	0.000	.182		LPA T1 → Total cholesterol T2	0.000	0.010	0.000	.794
Triglyceride T1 → LPA T2	17.547	0.054	27.459	.523		Total cholesterol T1 → LPA T2	6.642	0.083	6.391	.299
MVPA T1 → Triglyceride T2	0.000	−0.001	0.000	.973		MVPA T1 → Total cholesterol T2	−0.002	−0.051	0.001	.163
Triglyceride T1 → MVPA T2	−5.231	−0.025	16.688	.754		Total cholesterol T1 → MVPA T2	4.435	0.086	3.899	.255

Model was adjusted for baseline age, sex, insulin, triglyceride, high sensitivity C-reactive protein, heart rate, systolic blood pressure, glucose, fat mass, lean mass, smoking status, socioeconomic status, and family history of hypertension/diabetes/high cholesterol/vascular disease, and triglyceride, high-density lipoprotein cholesterol or low-density lipoprotein cholesterol depending on outcome with additional adjustment for sedentary time (ST), light physical activity (LPA) or moderate to vigorous physical activity (MVPA) depending on the predictor. Total cholesterol was not adjusted for lipids. Skewed variables were logarithmically transformed before analyses. A 2-sided *P*-value <.05 is considered statistically significant and are in bold.

Abbreviations: Time T1, 15 years of age; Time T2, 24 years; B, unstandardized regression; β, standardized regression, SE, standard error.

## Discussion

This longitudinal study demonstrates that poor movement behavior may be independently associated with poor lipid indices and that higher MVPA in adolescence may temporally precede lower low-density lipoprotein cholesterol in young adulthood. All movement behavior had paradoxical relationships with high-density lipoprotein cholesterol in which increased PA and less sedentary time were associated with decreased high-density lipoprotein cholesterol. Insulin resistance and body composition had differing mediating or suppressing roles in the associations of movement behavior with lipid indices, but low-grade inflammation had no significant effect. Total fat mass significantly attenuated the inverse associations of LPA and MVPA with low-density lipoprotein cholesterol and total cholesterol.

### Sedentary Time and Lipid Indices

The quality of evidence on the longitudinal associations of sedentary time with lipid indices was downgraded to very low because of the serious risk of bias and inconsistency, and short follow-up, hence the call for improved quality longitudinal evidence (7, 10, 37). In the present study, cumulative sedentary time was positively associated with cumulative low-density lipoprotein cholesterol and triglyceride even after mutual adjustments for LPA and MVPA, but there were no mediating effects of traditional risk factors such as fat mass and inflammation, and sedentary time–lipid relationships may not be temporal. These findings suggest that the contribution of sedentary time to lipid alterations may be more complex in growing children than previously identified in experimental studies (38-40). Importantly, insulin resistance significantly suppressed the relationship of sedentary time with low-density lipoprotein cholesterol and triglyceride. Sedentary time may decrease the expression of anti-inflammatory and antioxidative modulators such as nicotinamide N-methyltransferase as well as regulators of glucose transporter type 4 translocation (38, 40).

### Light Physical Activity and Lipid Indices

The latest World Health Organization PA guideline did not specifically mention LPA for children and adolescents (8). Longitudinal evidence on accelerometer-measured LPA in association with lipid indices is scarce in the pediatric population (6-8). In the present study, cumulative LPA was inversely associated with low-density lipoprotein cholesterol, triglyceride, and total cholesterol, and the relationship was significantly mediated by fat mass. This suggests that among children and adolescents with obesity or high-body fat mass, the effect of LPA in decreasing low-density lipoprotein cholesterol and triglyceride may decrease by nearly 30%. This is similar to a recent longitudinal report where fat mass decreased the effect of increased LPA on decreased inflammation by nearly 30% (11). Importantly, the strength of the associations of LPA with low-density lipoprotein cholesterol, triglyceride, and total cholesterol was 2- to 5-fold higher than the effect of MVPA on low-density lipoprotein cholesterol, triglyceride, and total cholesterol. Thus, LPA might be a pragmatic target for future interventions and public health guidelines in the pediatric population since it is more feasible and accessible, requires less motivation, incidental to daily living, and does not require a high level of exercise skill or prior fitness (3, 6, 7, 11, 41).

### Moderate to Vigorous Physical Activity and Lipid Indices

The relationships of MVPA with lipid indices in the pediatric population have been inconsistent in experimental and observational studies (6, 7). Observed positive MVPA effects on lipids levels during clinical trials tend to significantly attenuate after the intervention period (6). In the present study with a long observation period (13 years), cumulative MVPA from childhood was associated with cumulatively decreased low-density lipoprotein cholesterol, triglyceride, and total cholesterol that was suppressed by cumulative increase in lean mass but significantly mediated by increased total fat mass. It was observed that with the fat mass-mediating effect, the estimate of the inverse association of LPA with total cholesterol was decreased by 6.4% while the inverse association between MVPA and total cholesterol was decreased by 47.7%. While both LPA and MVPA were inversely associated with fat mass with a similar standardized effect estimate, an increased fat mass was associated with increased total cholesterol nearly 2 -fold when MVPA rather than LPA was the predictor. Furthermore, the standardized effect estimate of LPA inverse association with total cholesterol was at least 5-fold more than the standardized effect estimate of MVPA inverse association with total cholesterol. This suggests that LPA may have a better lipid-lowering effect than MVPA and that the MVPA effect can be significantly reduced by the presence of higher fat mass. It has been reported in children that higher fat mass predicted lower participation in exercise possibly due to low interest, comorbid conditions, and symptoms associated with vigorous exercise such as breathlessness, joint pains, muscle sprains, etc. (42). However, LPA provides an opportunity for persons with obesity to follow a path to potentially benefit from the lipid-lowering effect of mild exercise. This novel finding could explain the post-MVPA clinical trial failures in sustaining low lipid levels (6-8). Of note, the relationships between higher MVPA and lower low-density lipoprotein cholesterol may have potential causal inference, considering the consistent linear and temporal relationship. The clinical significance of this result is that a 1 mmol/L statin-induced reduction in low-density lipoprotein cholesterol was associated with a 20% decreased risk of major vascular events in adults (43). In the present study, a 60-minute MVPA from childhood may decrease low-density lipoprotein cholesterol by 0.12 mmol/L, which could approximate to a 2% risk reduction in major vascular events (43).

In the present study, it was observed that increased MVPA and LPA and reduced sedentary time were associated with decreased high-density lipoprotein cholesterol. It has been established that excessively elevated high-density lipoprotein cholesterol may be a sign of liver damage and has been associated with a higher risk of cardiovascular mortality in adults (44, 45). It is plausible that increased MVPA and LPA and decreased sedentary time promote liver health, enhancing optimal high-density lipoprotein cholesterol production and metabolism in this apparently healthy young population, but further experimental studies are warranted to explain the paradoxical associations of cumulative MVPA with decreased high-density lipoprotein cholesterol. It was also noted that increased lean mass suppressed the relationship between increased MVPA and decreased triglyceride. In the present study, an increase in lean mass was longitudinally associated with better lipid profiles, which might have been enhanced by higher PA level–induced improved oxidation capacity and mitochondrial function (46). Nonetheless, the benefits of MVPA on lipid indices may be significantly diminished by increased fat mass. The mediating effect of fat mass on the inverse associations of MVPA with low-density lipoprotein cholesterol among females was circa 11-fold larger (91% vs 8% mediation effect) than the mediating effect among males. Moreover, among females, the inverse associations of MVPA with low-density lipoprotein cholesterol was borderline significant. Plausible explanations could be the inherent female biology characterized by more body fat mass compared to males (20) in addition to females spending significantly less time in MVPA in relation to males (11).

### Strength and Limitation

The extensive array of gold standard and repeated measures of movement behaviors, body composition, and covariates throughout the follow-up period in the ALSPAC data set offered the possibility of using advanced statistical models to test the likelihood of reverse causality, temporality, and causal explanatory pathway. The autoregressive cross-lagged temporal path analyses examine the effect of 1 variable on another variable at a later time-point (47). The cross-lagged effects are adjusted for the effect of each variable at 1 time on the same variable later, which is the autoregressive effect, that represents the stability of each variable over time (47). Both cross-lagged and autoregressive effects are analyzed simultaneously allowing for examining temporal precedence (47). Temporality assessed with cross lagged analysis, which is a criterion for causal inference, is superior to cross-sectional correlational analysis (47). Moreover, the within-person level analyses may reflect potential causal effects than between-person associations (47). Observed or unobserved variables that are stable over time cannot, by design, confound within-level variables resulting in zero variations and correlations (47). This significantly reduces the risk for confounding and eliminates potential confounders bias (47). The findings fill knowledge gaps that might be useful in updating future PA guidelines (6-8, 10). Some limitations are that the study participants were mostly White, thus generalization of findings to other racial and ethnic groups is limited. Moreover, residual confounding such as the unavailability of fasting lipid indices at age 11 years, could bias the findings; however, lipid measures at age 17 years were included in the analysis. Cohort attrition could lead to bias in observational studies, which may be negligible since participants who lacked certain movement behavior and metabolic variables had similar characteristics to those included in the analyses. The current study lacked data on the participants’ dietary habits, which could be a source of residual bias; however, it is known that body composition and metabolic indices reflect the participants’ diet. Moreover, accounting for total energy intake in early adolescence did not significantly alter the results (data not shown). Other unmeasured factors such as alcohol intake and menstrual cycle may bias the results, and future studies may account for these covariates. The accelerometer data were collected using a 60-second epoch that is known to underestimate MVPA in pediatric populations.

### Conclusion

Increasing cumulative MVPA may temporally precede decreased low-density lipoprotein cholesterol only but be associated with decreased total cholesterol. Cumulative sedentary time was associated with increasing low-density lipoprotein cholesterol, triglyceride, and total cholesterol, while LPA was associated with decreasing low-density lipoprotein cholesterol, triglyceride, and total cholesterol, but the relationship may not be temporal. Promoting LPA and MVPA while decreasing sedentary time may be considered crucial intervention targets to attenuate the risk of elevated lipid levels and their sequelae in the pediatric population. Increased LPA had a 5- to 8-fold total cholesterol-lowering effect and was more resistant to the attenuating effect of fat mass compared with MVPA.

## Data Availability

The informed consent obtained from ALSPAC participants does not allow the data to be made freely available through any third-party–maintained public repository. However, data used for this submission can be made available on request to the ALSPAC Executive. The ALSPAC data management plan describes in detail the policy regarding data sharing, which is through a system of managed open access. Full instructions for applying for data access can be found here: http://www.bristol.ac.uk/alspac/researchers/access/. The ALSPAC study website contains details of all the data that are available (http://www.bristol.ac.uk/alspac/researchers/our-data/).

## Abbreviations

LPA: light physical activity
MVPA: moderate to vigorous physical activity
PA: physical activity
